# Rape and Its Penalty

**Published:** 1904-10

**Authors:** 


### *EDITORIAL NOTES AND COMMENTS.*

The Business office of THE JOURNAL-RECORD is No. 1007-1008 Century Building.

The Editorial office is 318-319-320 The Prudential.

Address all Business communications to Dr. M. B. Hutchins, Mgr.

Make remittances payable to THE ATLANTA JOURNAL-RECORD OF MEDICINE.

On matters pertaining to the Editorial and Original Communications address Dr. Bernard Wolff, Atlanta.

Reprints of original articles will be furnished at cost price. Requests for the same should always be made *on the manuscript.*

We will present, post-paid, on request, to each contributor of an original article, twenty (20) marked copies of THE JOURNAL-RECORD containing such article.

### RAPE AND ITS PENALTY.

SINCE the recent recrudescence of the lynching spirit, the newspapers have been devoting much space to inquiries into the proper methods to pursue to put a stop to the practice. The trend of opinion seems to be expressed in the phrase, " Make the law stronger in order to stop crime." In other words, "Recourse to the law is the proper corrective." "Though justice moves with leaden feet, she strikes with an iron hand"—much in the same way as slapping at a mosquito—a slow, cautious lifting of the hand, then a vehement and vengeful slap—hope, anticipation, while the hand is turned over, what does it reveal? Nothing.

In the terminal dissection of the subject, does the catch-phrase first quoted solve the question even in part? Law is a mere convention—an intricate set of rules formulated from accumulated human experience and based on the Golden Rule. But in itself the question of lynching is not a question of law but of lawlessness. Be the law lax or stringent no thought is given it in the mob's wild *dêbacle* of revenge. Respect and familiarity with the law leads one to overestimate its efficiency as a repressant of crime—not of crime in general but of the specific, the master crime of rape. Did ever law stay the ravisher's hand from the commission of the foul deed upon the defenceless woman on the lonely farm? If dread of the swift and sure vengeance of the mob with torture and the stake does not turn him aside from his sinister purpose, will the sombre tragi-farce of the county court and the shadowy emblem of legal execution looming beyond make him hesitate?

What is law to a skulking satyr, far from the intruding world, aflame with primitive passions, scorning danger, ignoring the terrible retributive justice that inexorably awaits him, though he can almost hear the crackling of the flames about him or knows that in a short time his riddled body will swing from a limb like fruit in King Louis' orchard close. Law as a punishment for crime and law as a preventive of crime are distinct propositions. The former attaches penalties, the latter seeks to repress by fear of executing the penalties prescribed. But should the fear of punishment be absent in the potential or actual criminal the awesome majesty of the law is but the tawny skin that fails to conceal the hairy ears of the jackass.

No, it is obvious that the notable ineffectiveness of the law to deal with the various phases of the lynching problem, must lead us behind and beyond it for solution and redress.

How can the crime of rape be prevented and lynching suppressed? This is a question that has vexed the public mind for decades, and the answers have been many, some revolutionary, some utopian, some practical. The castration of rapists is assuredly *argumentum ad hominem.* It is certain that whatever might be the subsequent fate of the criminal thus dealt with, strict and inviolable chastity would be thereafter his rule of conduct. But it is *ex post facto.* Nero wished that all Rome had but one neck so that all heads might be severed at one blow. A similar wish might be entertained for a composite set of organs—but *verbum sap*. The plan has its merits. It annihilates the function that actuates the deed. But the effect, though impressive, is not sufficiently diffusive. It is too sporadic and isolated. It will not cure the evil.

John Temple Graves advocates the wholesale deportation of negroes and their sequestration in a state of their own, where their evolution and progress, which, according to certain quasi-philanthropists, is so hampered by white environment and competition, may expand and flourish untrammeled. It would be an interesting experiment, but how successful the fate of Hayti and San Domingo must rise to view as a forecast.

For a poison to be remedially effective it must be diluted to the point where it loses its toxicity. It is thus with the black botch which has withered the South. Crude, raw and congested the hordes of negroes in the South bulk in a huge shadow. Scatter the mass by permeation with white labor from the overcrowded countries of Europe or of Asia. In the latter case is a yellow peril more to be dreaded than a black actuality ?

Black labor is time-honored in the South but is not indispensable. It is the general verdict that it can be eliminated without disaster and with only temporary inconvenience. This explanation, with much hesitation and more modesty, is offered as the solution of the negro problem and with it the crime of rape and of lynching.

The change will be gradual. It can not be effected at once. But so surely as the emptiness of the South is filled by white people, the negro problem will be settled. Spread broad and thin, he will cease to be an object of interest. And when he is viewed with indifference and is no longer the bone of contention, held in an artificial position by contending forces, placed fairly and squarely on the level of competition, his fate will be sad indeed.

